# Formation of Hydrogels Based on a Copolymer of *N*-Vinyl-2-pyrrolidone and Glycidyl Methacrylate in the Presence of the Reaction Product of 1,3-Dimethylmidazolium Dimethylphosphate and Elemental Sulfur

**DOI:** 10.3390/gels8020136

**Published:** 2022-02-21

**Authors:** Natalia Tarasova, Efrem Krivoborodov, Alexey Zanin, Ekaterina Pascal, Ilya Toropygin, Alexander Artyukhov, Samson Muradyan, Yaroslav Mezhuev

**Affiliations:** 1 Institute of Chemistry and Problems of Sustainable Development, Dmitry Mendeleev University of Chemical Technology of Russia, 125047 Moscow, Russia; tarasnp@muctr.ru (N.T.); vv1992@yandex.ru (E.K.); kate.nibbler@gmail.com (E.P.); artyukhow@yandex.ru (A.A.); ololomg16@gmail.com (S.M.); valsorja@mail.ru (Y.M.); 2 Institute of Geology of Ore Deposits, Petrography, Mineralogy, and Geochemistry, Russian Academy of Sciences, 119017 Moscow, Russia; 3 V.N. Orekhovich Research Institute of Biomedical Chemistry, Russian Academy of Medical Sciences, 119121 Moscow, Russia; toropygin@rambler.ru

**Keywords:** PVP hydrogels, “one-pot” synthesis, swelling kinetics, medical hydrogels, *N*-vinyl-2-pyrrolidone, glycidyl methacrylate, ionic liquids, sulfur

## Abstract

The aim of the study is to search for a reaction that provides the possibility of tandem “one-pot” formation of polymer networks during radical copolymerization of *N*-vinyl-2-pyrrolidone and glycidyl methacrylate. It was shown that the addition of recently synthesized 1,3-dimethylimidazolium (phosphonooxy-)oligosulfanide makes it possible to obtain a cross-linked copolymer in one stage as a result of radical copolymerization of *N*-vinyl-2-pyrrolidone and glycidyl methacrylate with a molar ratio of monomers less than 1.4. The structure of the copolymerization products of *N*-vinyl-2-pyrroldione and glycidyl methacrylate formed in the presence of 1,3-dimethylimidazolium (phosphonooxy-)oligosulfanide was characterized by ^1^H NMR, FTIR and MALDI spectroscopy. ^1^H NMR spectroscopy revealed an interaction under moderate heating between glycidyl methacrylate and 1,3-dimethylimidazolium (phosphonooxy-)oligosulfanide, accompanied by the formation of a mixture of unsaturated products of complex structure, presumably acting as crosslinking agents. It is shown that when the molar ratio of *N*-vinyl-2-pyrroldione/glycidyl methacrylate comonomers is 0.89, a densely crosslinked copolymer is formed, capable of limited swelling in water with a velocity constant of 5.06 × 10^−2^ min^−1^ and an equilibrium degree of swelling of about 227%.

## 1. Introduction

Poly(*N*-vinyl-2-pyrrolidone) is one of the water-soluble polymers promising for the creation of adhesive compositions [1], wastewater treatment systems [2,3] and drug delivery [4,5,6]. Poly(*N*-vinyl-2-pyrrolidone) is also used to impart hydrophilic properties to fibers [7,8], as a modifier and pore-forming agent in the preparation of membranes [8,9,10,11], as well as for the formation of nanoparticles [12,13] and hydrogels [14]. Although poly(*N*-vinyl-2-pyrrolidone)-based hydrogels are promising for the inclusion of drugs and water treatment [6], the chemical inertia of *N*-vinyl-2-pyrrolidone residues [5,6] creates impediments for crosslinking chains in one stage under mild conditions. Crosslinking of poly(*N*-vinyl-2-pyrrolidone) chains can be carried out as a result of exposure to radiation [15,16,17], oxidation with potassium persulfate [18], copolymerization with monomers containing more than one double bond [19,20], or as a result of specific interactions with chains of other water-soluble polymers [21,22]. At the same time, the crosslinking of poly(*N*-vinyl-2-pyrrolidone) under the influence of high-energy radiation can lead to the parallel development of the radiation destruction process, and the use of polyunsaturated crosslinking agents creates difficulties in cleaning hydrogels. Another approach to the crosslinking of chains and the formation of hydrogels is the copolymerization of *N*-vinyl-2-pyrrolidone with monomers containing reactive functional groups, for example, epoxy group [23]. In this case, the formation of hydrogels containing hydrophilic residues of *N*-vinyl-2-pyrrolidone includes the stages of obtaining functionalized copolymers and their crosslinking.

**Scheme 1:**
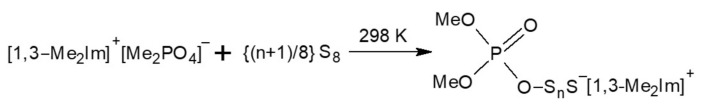
Interaction of 1,3-dimethylimidazolium dimethylphosphate with elemental sulfur. Adapted from [28,29].

The last 30 years have been marked by an intensive search for new tandem reactions that allow the realization of sequences of two or more stages in one reaction [24]. Therefore, the purpose of this article is to search for a principle scheme that provides the possibility of forming polymer networks under mild conditions in one stage during copolymerization of *N*-vinyl-2-pyrrolidone and a model epoxy group containing monomer—glycidyl methacrylate. Although the copolymerization of *N*-vinyl-2-pyrrolidone and glycidyl methacrylate was described earlier, the resulting copolymers have a linear structure [25,26] and subsequent crosslinking is required to form a polymer network [27]. At the same time, the authors have recently discovered the possibility of opening the S8 ring under exceptionally mild conditions in the presence of dimethyl phosphate-containing ionic liquids [28,29] as a result of the reaction shown in Scheme 1.

The resulting 1,3-dimethylimidazolium (phosphonooxy-)oligosulfanide (POOS) exhibits pronounced nucleophilic properties and is capable of initiating anionic polymerization of formaldehyde [30] and ethyl 2-cyanoacrylate [31] under green conditions. This article shows the possibility of using the high nucleophilicity of POOS to open the epoxy ring of glycidyl methacrylate, which opens up an approach to the tandem “one-pot” synthesis of polymer networks based on *N*-vinyl-2-pyrrolidone copolymers.

## 2. Results and Discussion

### 2.1. Interaction between GMA and POOS

**Figure 1:**
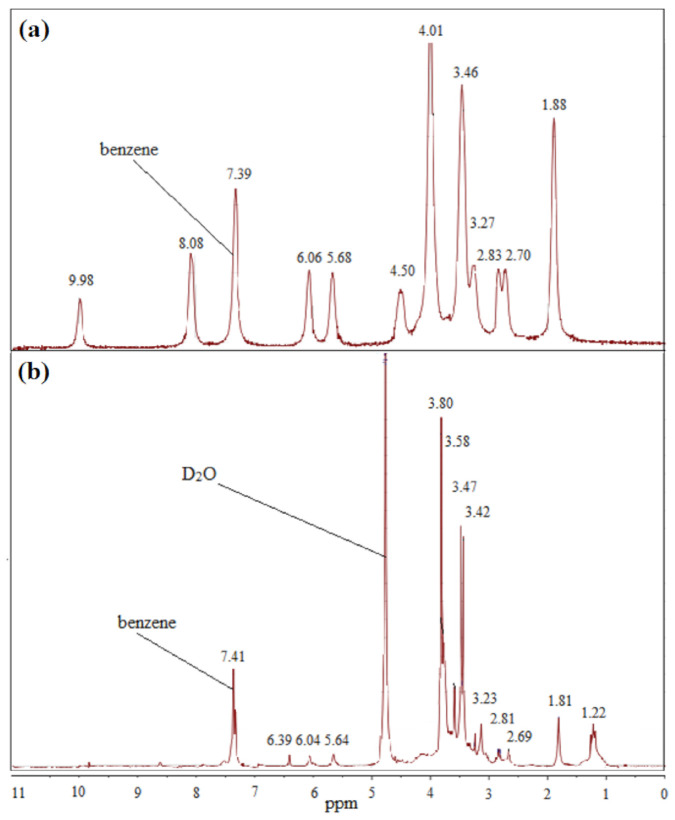
NMR ^1^H spectra of a mixture of GMA and POOS at temperatures: (**a**)—298 K; (**b**)—323 K after holding for 8 h (with the addition of D_2_O when registering the NMR ^1^H spectrum).

Since three-dimensional polymer networks are formed as a result of the “one-pot” radical copolymerization of VP and GMA initiated by Azobisisobutyronitrile (AIBN) in the presence of POOS at 348 K, the reaction of GMA and POOS (without VP) was investigated at first. It was found that at a temperature of 298 K, the chemical interaction of GMA and POOS does not occur at a noticeable rate, and the ^1^H NMR spectrum of the reaction system represents a superposition of the spectra of each of the substances (Figure 1a). However, when the temperature rises to 323 K, a chemical reaction occurs, leading to the formation of a highly viscous dark red liquid. A new doublet of 1.22 m.d. appears in the ^1^H NMR spectrum, indicating the formation of a methyl group in the vicinity of a saturated fragment (Figure 1b). The intensity of the signals of the geminal protons of the GMA double bond, manifested in the region of chemical shifts of 5.5–6.1 m.d., significantly decreases, while the chemical shift and the intensity of the protons of the glycidyl fragment also changes (Figure 1b). Consequently, the reaction of POOS with GMA affects both the double bond and the epoxy group.

**Scheme 2:**
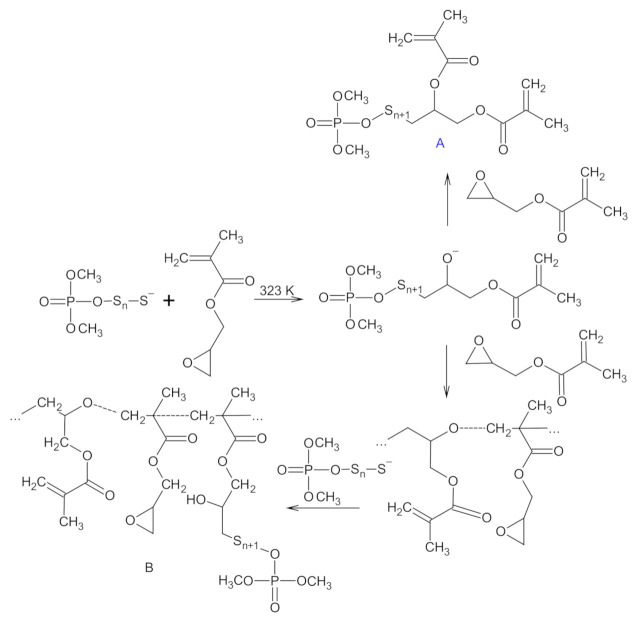
Interaction of (phosphonooxy-)oligosulfanide-anion with GMA.

Although POOS proved to be an effective initiator of anionic polymerization of formaldehyde [30] and ethyl 2-cyanoacrylate [31], the less electrophilic monomer methyl methacrylate did not polymerize. Consequently, the conjugated nucleophilic addition of POOS to the electron-deficient GMA double bond does not seem to be the main direction of the reaction. However, the appearance of a doublet with a chemical shift of 1.22 ppm (Figure 1b) indicates the addition of a nucleophile other than POOS to the double bond of the methacrylate group GMA. Therefore, it can be assumed that the interaction of POOS with GMA begins with the opening of the epoxy ring and the formation of an alcoholate anion capable of joining via the GMA double bond or causing transesterification (Scheme 2). The sequence of reactions shown in Scheme 2 leads to the formation of a mixture of macromeres in the reaction system that perform the function of crosslinking agents, the structure of which is apparently close to A and B.

### 2.2. Synthesis and Characterization of VP and GMA Copolymers Obtained in the Presence of POOS

**Scheme 3:**
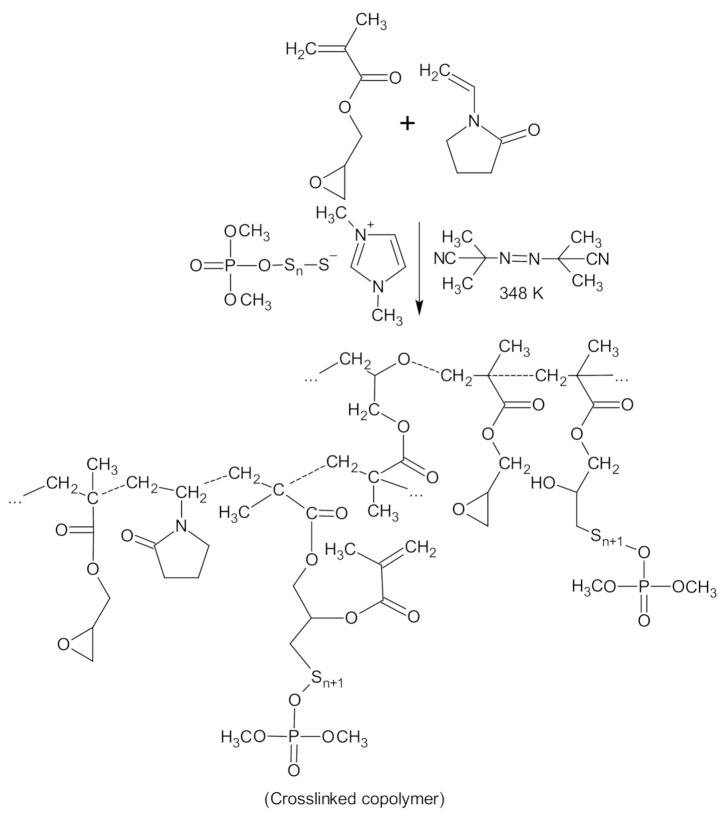
Polymer network formation during radical copolymerization of VP and GMA initiated by AIBN in the presence of POOS.

As can be seen, the reaction of the POOS anion with GMA, occurring at a temperature of 323 K, leads to the formation of unsaturated monomers A and B (Scheme 2). This reaction is all the more possible at a temperature of 348 K, which provides a decay rate of AIBN sufficient to initiate radical copolymerization. Thus, along with GMA and VP at a temperature of 348 K, crosslinking agents A and B are present in the system, formed as a result of the reaction of GMA and the POOS anion. Apparently, radical copolymerization of *N*-vinyl-2-pyrrolidone with macromeres A and B formed in the reaction system is the reason for the formation of polymer networks. Thus, the reaction product (Scheme 3) is a polymer with an irregular structure containing residues of *N*-vinyl-2-pyrrolidone, glycidyl methacrylate, as well as branching chain units, such as residues of macromeres A, B, and related structures. In addition, after the formation of the gel, some of the unsaturated fragments are preserved, which is due to their physical isolation. Therefore, the structure of the cross-linked copolymer shown in Diagram 3 should be considered to be conditional. 

To determine the ratio of comonomers at which the formation of a polymer network is observed, copolymerization of various amounts of VP and GMA was carried out (Table 1). It is established that the formation of crosslinked polymers is observed only if the molar ratio VP/GMA in the reaction mixture is less than 1.4. When the molar ratio VP/GMA increases above 1.4, products are formed that swell indefinitely in water, which opens up the possibility of registering their NMR ^1^H spectra in the D_2_O medium.

**Figure 2:**
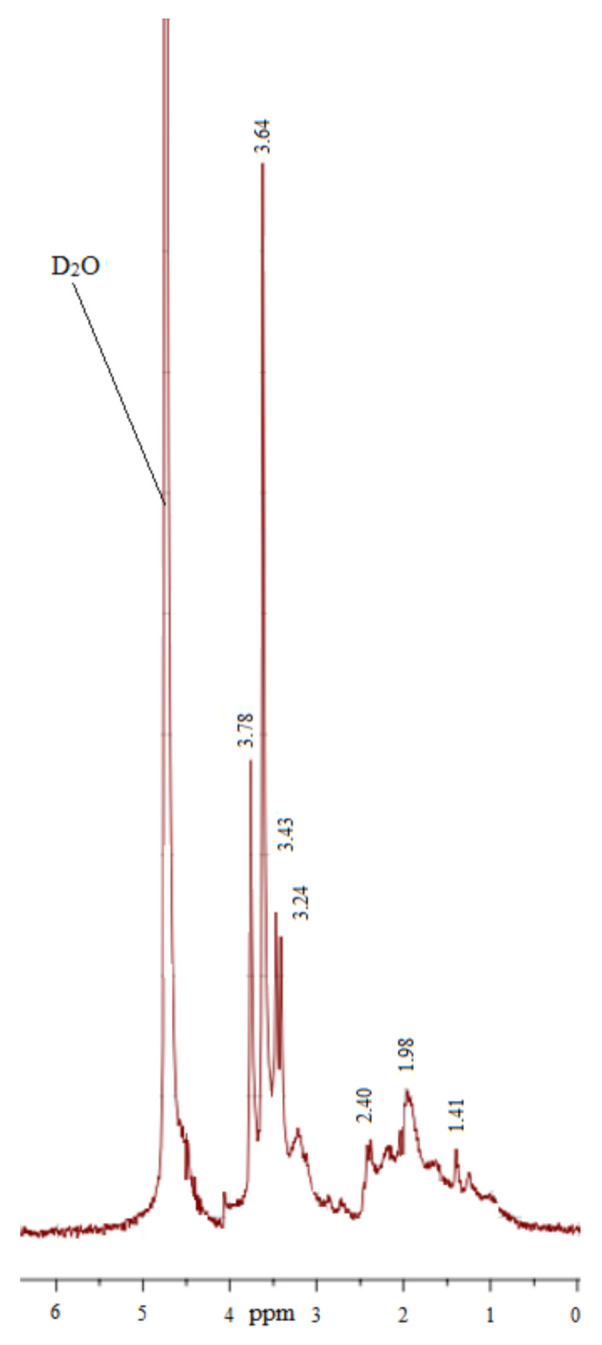
NMR ^1^H spectrum in D_2_O medium registered for a copolymer obtained at a molar ratio VP/GMA equal to 1.4.

As can be seen, the NMR ^1^H spectrum of the product of radical copolymerization of VP and GMA in the presence of POOS (Figure 2) has a complex character, and there are no signals characteristic of double bonds [32]. Thus, copolymerization of *N*-vinyl-2-pyrrolidone with macromeres A and B occurs in unsaturated fragments, and the resulting copolymer has a complicated architecture, which is consistent with the mechanism shown in Scheme 2.

**Figure 3:**
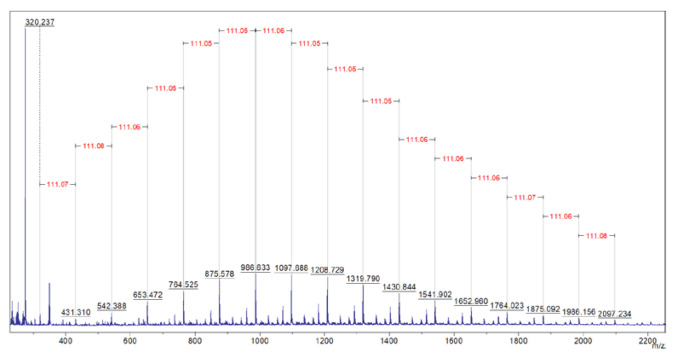
MALDI-TOF is the spectrum of a copolymer synthesized at a molar ratio of VP/GMA equal to 1.4, registered under conditions of laser desorption with a DHB matrix.

The MALDI spectrum recorded for a sample of a copolymer synthesized at a molar ratio of VP/GMA equal to 1.4 is shown in Figure 3. Small values of molecular weights and the presence of only signals of chains constructed by VP residues indicate the realization of macromolecule destruction under laser desorption conditions. It is obvious that fragments of chains containing GMA residues, which perform the function of chain branching points, have less volatility, therefore, during laser desorption, signals are observed only in fragments released due to destruction, constructed by VP residues. 

**Figure 4:**
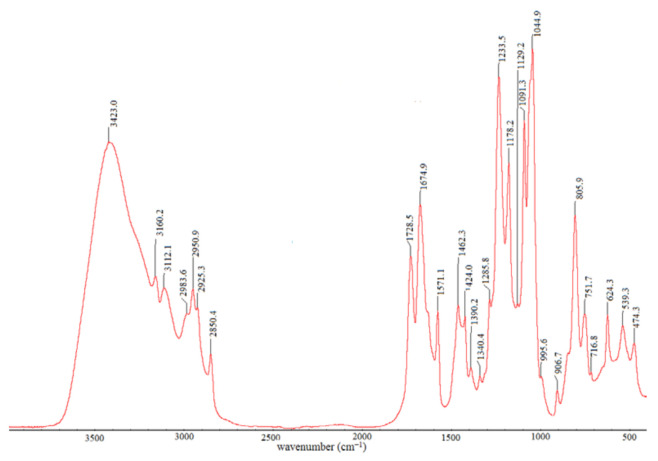
FTIR spectrum of a copolymer synthesized at a molar ratio of VP/GMA in the reaction system 0.89, registered in potassium bromide tablets.

If the molar ratio of VP/GMA in the reaction system is 0.89, then the formation of a polymer mesh capable of limited swelling in water is observed. Bands with wave numbers 1728.5 cm^−1^ and 1674.9 cm^−1^ correspond to valence vibrations of carbonyl groups of GMA and VP residues in the polymer chain, respectively [33] (Figure 4).

The band caused by valence vibrations of double C=C bonds manifests itself in the region of 1630–1665 cm^−1^ and is partially blocked by the absorption of the amide carbonyl of VP. The presence of residual double bonds is indicated by the bands 906.7 cm^−1^ and 995.6 cm^−1^, corresponding to out-of-plane and planar deformation vibrations of C−H with double bonds. Thus, in the formation of three-dimensional polymer networks, the residual amount of double bonds is apparently greater than in the formation of branched, but still water-soluble copolymers. This can be explained by the isolation of a part of the double bonds as a result of the loss of fluidity by the reaction system after the formation of the polymer networks.

Absorption bands typical of epoxides are absent in the FTIR spectrum of the crosslinked copolymer, which confirms their participation in the formation of the polymer mesh. At the same time, there is a wide intense signal of 3423 cm^−1^ corresponding to the valence vibrations of O–H bonds formed as a result of the opening of the epoxy ring.

**Figure 5:**
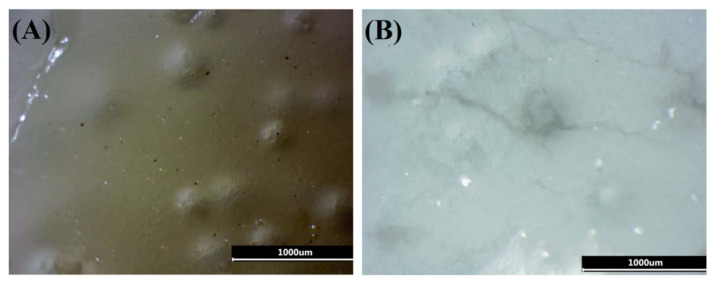
Photos of the surfaces of the crosslinked polymer before swelling in water (**A**) and after reaching the equilibrium degree of swelling in water (**B**).

Photos of the surface of the copolymer obtained at the molar ratio VP/GMA in the reaction system 0.89 and the same sample, which has reached an equilibrium degree of swelling, are shown in Figure 5. As can be seen, the formed hydrogel is opaque. 

### 2.3. Swelling Kinetics and Crosslink Density

**Figure 6:**
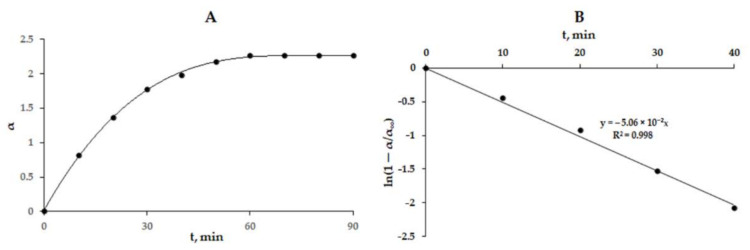
Kinetic curves of swelling of the crosslinked polymer in water in coordinates: (**A**)—“vs. t” (**B**)—“ $\ln\left[1-\frac{\alpha }{\alpha_{\infty }}\right]\mathrm{vs}.t$ ” (the cross-linked copolymer was synthesized at the molar ratio VP/GMA in the reaction system 0.89).

When the equilibrium degree of swelling is reached ($\alpha_{\infty }=2.267$), the mass of the sample increases by 3.26 times (Figure 6A). The kinetics of swelling of the cross-linked copolymer in water at a temperature of 298 K obeys the first-order Equation (1) [34] with a velocity constant of 5.06 = 10^−2^ min^−1^ (Figure 6B).

(1) $$\ln\;\left[1-\frac{\alpha }{\alpha_{\infty }}\right]\;=-kt$$

where: $\alpha ,\;\alpha_{\infty }$—the degree of swelling and the equilibrium degree of swelling of the polyacrylamide gel in water; t—time; k—swelling rate constant. 

The average molecular weight of the chain segments between the grid nodes can be calculated in accordance with the theory of Flory and Rehner by Equation (2) [34,35].

(2) $${\bar{M}}_{c}=\;-\;\frac{d_{P}V_{mS}\left(\varphi^{\frac{1}{3}}-0.5\varphi \right)}{\ln\left(1-\varphi \right)\;+\;\varphi \;+\;\chi \varphi^{2}}$$

where: ${\bar{M}}_{c}$—average molecular weight of chain segments between network nodes; φ—volume fraction of the polymer that has reached equilibrium swelling; $d_{P}$—density of the polymer; $V_{mS}$—molar volume of the solvent; χ—Huggins interaction parameter.

Since the gel formed after removal of 1,4-dioxane contains pores, some of which can be closed, the density was estimated as a linear function of the composition of the resulting cross-linked copolymer. Taking into account the calculated sample density of about 1.164 g·cm^3^, the volume fraction of the crosslinked polymer, when equilibrium swelling is reached, is about 0.264. Taking the Huggins interaction parameter as equal to 0.489 [16], the value of ${\bar{M}}_{c}$, calculated by Equation (2) is about 1.26 × 10^3^. Consequently, the resulting crosslinked polymers have a high crosslinking density, which can explain the relatively small value of the equilibrium degree of swelling, which is about 227%. A significant cross-linking density ensures sufficient rigidity of the cross-linked polymer sample after removal of 1,4-dioxane, as well as maintaining the continuity of the gel when swelling in water, taking into account the known ability of VP residues in polymer chains to non-covalent binding of polar organic compounds, the resulting cross-linked polymer can be recommended for use as a sorbent.

## 3. Conclusions

A new approach to the “one-pot” formation of gels as a result of radical copolymerization of *N*-vinyl-2-pyrrolidone and glycidyl methacrylate in the presence of 1,3-dimethylimidazolium (phosphonooxy-)oligosulfanide has been developed. It was found that the polymer network is formed at a molar ratio of *N*-vinyl-2-pyrrolidone/glycidyl methacrylate less than 1.4. The reason for the formation of the polymer network is the interaction of 1,3-dimethylimidazolium (phosphonooxy-)oligosulfanide with glycidyl methacrylate, leading to the formation of a complicated mixture of unsaturated products capable of copolymerization with *N*-vinyl-2-pyrrolidone. Polymer networks formed with a molar ratio of *N*-vinyl-2-pyrrolidone/glycidyl methacrylate equal to 0.89 have a significant crosslinking density (${\bar{M}}_{c}$ = 1.26 × 10^3^) and are capable of limited swelling in water with a 3.26-fold increase in mass. The obtained hydrogels are of interest as sorbents for water purification from dissolved organic compounds, as well as for use as the basis of wound dressings, providing sorption of exudate.

## 4. Materials and Methods

### 4.1. Materials

*N*-vinyl-2-pyrrolidone, glycidyl methacrylate, 1,3-dimethylimidazolium dimethylphosphate, azobisisobutyronitrile (AIBN), 1,4-dioxane were purchased from “Sigma–Aldrich” (St. Louis, MO, USA). Elemental sulfur was purchased from “Reakhim” (Samara Oblast, Russia). Synthesis of polymers under the influence of microwave radiation was carried out using a microwave reactor “Biotage initiator+”. 

### 4.2. Structure Characterization Methods

IR spectra of the samples were recorded in potassium bromide pellets using a Nicolet 380 spectrometer (Thermo Fisher Scientific, Waltham, MA, USA). ^1^H NMR spectra were registered on Bruker CXP 200, Chemical shifts in ^1^H spectra were measured relative to the signal of (CH_3_)_4_Si. Micro photo images were processed using the MCview (Lomo Microsystems, St-Petersburg, Russia) software package via Lomo MSP-2-2SD stereoscopic microscope equipped with an MC-5 electronic camera (Lomo Microsystems, Russia). MALDI spectra were recorded on an Ultraflex II mass spectrometer (Bruker, Karlsruhe, Germany) with an accelerating voltage of 25 kV with Nd: YAG laser (355 nm) desorption from a DHB matrix.

### 4.3. Reaction of GMA and POOS

0.3 mL of POOS (synthesized according to the previously described method [28,29,30,31,34]) was added to 0.2 mL of GMA, then the reaction mixture was stirred for 60 min at room temperature until the color change of the sample stopped. Then the prepared GMA-POOS solution was heated in a thermostat at 323 K for 8 h. The structure of the resulting product was characterized by ^1^H NMR spectroscopy.

### 4.4. Synthesis of the VP-GMA-POOS Copolymers

To determine the ratio of monomers leading to the formation of polymer networks, copolymerization of various amounts of *N*-vinyl-2-pyrrolidone and GMA was carried out. In a vial, known amounts of VP and a solution containing GMA (40 vol%) and POOS in benzene (60 vol%) were dissolved in 0.5 mL of 1,4-dioxane (Table 1). Copolymerization was carried out in a microwave reactor at a temperature of 348 K for 150 min. AIBN in the amount of 10 mol% of VP was used to initiate radical copolymerization.

### 4.5. Testing the Swelling Kinetics

0.12 g of the copolymer obtained with a volume fraction of VP in the reaction system of 30% was placed in 25 mL of water. The kinetics of swelling was studied by recording the values of the sample masses to the specified time points at a temperature of 298 K.

## Figures and Tables

**Table 1:** Parameters of sample synthesis.

Number of Item/ Components	1	2	3	4	5	6
GMA-POOS, vol%	80	70	60	50	40	30
VP (ml), vol%	20	30	40	50	60	70

## Data Availability

The data presented in this study are available on request from the corresponding author.
